# Identification of KCNQ1 as a diagnostic biomarker related to endoplasmic reticulum stress for intervertebral disc degeneration based on machine learning and experimental evidence

**DOI:** 10.1097/MD.0000000000040661

**Published:** 2024-11-29

**Authors:** Feng Wu, Xin Hu, Xing Li, Yongquan Huang

**Affiliations:** a Department of Orthopaedics, Pingxiang People’s Hospital, Pingxiang, Jiangxi, China.

**Keywords:** biomarker, endoplasmic reticulum stress, intervertebral disc degeneration, KCNQ1, machine learning

## Abstract

Intervertebral disc degeneration (IDD) is a primary cause of low back pain and disability. Cellular senescence and apoptosis due to endoplasmic reticulum stress (ERS) are key in IDD pathology. Identifying biomarkers linked to ERS in IDD is crucial for diagnosis and treatment. We utilized machine learning on gene expression profiles from the Gene Expression Omnibus database to discover biomarkers associated with ERS in IDD. Gene set enrichment analysis (GSEA) and single-sample GSEA were applied to evaluate the immunological features and biological functions of these biomarkers. The expression of KCNQ1 was experimentally validated. Machine learning identified KCNQ1 as a diagnostic biomarker for ERS in IDD, confirmed by Western blotting. GSEA indicated that KCNQ1 influences IDD primarily through the Notch signaling pathway and by regulating macrophage and monocyte infiltration. KCNQ1, identified as an ERS-associated biomarker in IDD, impacts the Notch signaling pathway and immune cell infiltration, suggesting its potential as a therapeutic target for IDD. Further validation through prospective studies and additional experimental methods is necessary to elucidate the role of KCNQ1 in IDD comprehensively.

## 1. Introduction

Low back pain is widely recognized as the primary cause of disability, resulting in significant mental stress and financial burden for people.^[1,2]^ Among the most common reasons for low back pain, intervertebral disc degeneration (IDD) is a well-known culprit.^[3–5]^ The evolution of IDD can result in the development of spinal degenerative conditions such as disc herniation, spinal canal stenosis, spondylolisthesis, and degenerative scoliosis. These conditions can cause significant pain and impairment.^[6]^ Presently, a multitude of therapeutic approaches exist for the management of IDD, primarily encompassing pharmacological intervention and surgical intervention. Nevertheless, the majority of these solutions are subject to constraints.^[7,8]^ Despite some research on IDD in recent years, the fundamental mechanisms are still not well understood.^[9]^ Hence, it is imperative to investigate the fundamental mechanisms and devise innovative therapeutic strategies for the prompt recuperation of IDD.

The endoplasmic reticulum (ER) is crucial for preserving the proper physiological structure and carrying out physiological tasks in the intervertebral disc.^[10,11]^ Endoplasmic reticulum stress (ERS) occurs when there is a buildup of proteins that are not properly folded or unfolded in the ER, resulting from a deficiency in the ER’s ability to fold proteins. This has a harmful impact on the normal functioning and viability of various types of cells.^[12]^ Several studies have demonstrated that ERS is linked to the development of IDD through its involvement in oxidative stress, disruption of calcium homeostasis, inflammatory reactions, excessive mechanical loading, and metabolic dysfunction.^[13–16]^ Overall, ERS induced cellular aging and apoptosis play an important role in inducing pathological features of IDD.^[17]^ Hence, the identification of diagnostic biomarkers and their functions associated with ERS in IDD is crucial for the identification and management of IDD. Nevertheless, there have been no reported diagnostic biomarkers of significance thus far. Hence, our research aim is to identify potential biomarkers associated with ERS in IDD, offering novel insights and avenues for the diagnosis and treatment of IDD.

The difficulty in biomarker discovery rests in successfully accomplishing feature selection, which is a critical task in this field.^[18]^ Fortunately, the problem can be efficiently resolved via machine learning (ML). ML defines the ability of a machine to learn and predict future events and outcomes based on large datasets.^[19]^ Presently, numerous studies have employed ML techniques to scrutinize diagnostic biomarkers for diseases.^[20–22]^ In this study, we screened for ERS related diagnostic biomarkers in IDD using 3 popular ML algorithms: least absolute shrinkage and selection operator (LASSO), support vector machine-recursive feature elimination (SVM-RFE) and random forest (RF). As a normalized linear regression method, LASSO regression can discard unnecessary features and produce a sparse and easy-to-interpret model to avoid overfitting.^[23]^ SVM-REF is capable of selectively extracting pertinent features and eliminating relatively insignificant feature variables in order to enhance classification accuracy.^[24]^ Random forest (RF), created by Breiman, is a technique for ensemble learning that combines several predictions to decrease variability and enhance the reliability and precision of the output.^[25]^ Through the use of the aforementioned 3 ML methods, we successfully determined KCNQ1 as the most effective diagnostic biomarker linked to ERS in IDD. Furthermore, we conducted functional analysis using gene set enrichment analysis (GSEA). Simultaneously, we performed immune cell infiltration analysis to thoroughly examine the mechanism by which KCNQ1 operates in IDD.

## 2. Methods and materials

### 2.1. Downloading and preparing the datasets

The gene expression profile data were acquired from the Gene Expression Omnibus through a search for RNA-seq profiles using the phrase “intervertebral disc degeneration.” Three datasets were identified: the training dataset (GSE150408^[26,27]^) and 2 validation datasets (GSE56081^[28,29]^ and GSE70362^[20,30]^). GSE150408 consisted of 17 individuals with IDD and 17 individuals without IDD, serving as normal controls. The validation datasets comprised GSE56081, which included 5 patients with IDD and 5 controls, and GSE70362, which included 16 patients with IDD and 8 normal controls. The data underwent background correction and normalization using the R programme “limma.” Due to the use of publicly available data and the absence of any experimental interventions requiring such review, this study does not require ethical approval.

### 2.2. Identification of ERS related DEGs in IDD

In this study, a gene set comprising 785 genes associated with ERS was obtained from GeneCards (www.genecards.org) and employed as part of our methodology. The process of distinguishing ERS related differentially expressed genes (DEGs) between samples obtained from healthy controls and patients was carried out using the “limma” package in R. A screening criterion of a Wilcoxon test and a *P* value threshold of <.01 were employed for this purpose.

### 2.3. GO and KEGG enrichment analysis of DEGs

The correlation analysis between DEGs was conducted utilizing the “corrplot” utility in R. In order to examine extensive information pertaining to gene data on a large scale, prevalent bioinformatic techniques including KEGG pathway enrichment analysis^[31]^ (https://www.kegg.jp) and gene ontology (GO) enrichment analysis were utilized. The outcomes of these analyses were represented graphically through the utilization of the “GOplot” software application.

### 2.4. Diagnostic biomarkers screening with ML

Three ML techniques were employed to identify diagnostic biomarkers of IDD linked to ERS: LASSO, SVM-RFE, and RF. The packages, “glmnet,” “e1071” and “RandomForest” were utilized to perform LASSO, SVM, and RF, respectively. Utilizing Venn diagrams, determine the most effective diagnostic biomarker associated with ERS in IDD.

### 2.5. Development and verification of a diagnostic biomarker

The statistical significance of the disparity in diagnostic biomarkers between the disease and control groups was assessed using a significance threshold of *P* < .05. The predictive capability of the potential diagnostic biomarker was then evaluated in datasets using receiver operating characteristic (ROC) analysis, employing the “pROC” package.

### 2.6. Gene set enrichment analysis (GSEA) of the diagnostic biomarker

To further investigate the possible role of diagnostic biomarkers in IDD, GSEA was employed to identify functional categories and pathways enriched for diagnostic biomarkers. To perform GSEA, the R package “clusterprofiler” was used. The Molecular Signatures Database provided the reference gene set, h.all.v6.2.sytmbols.gmt, and the cutoff value was *P* adjusted value .05.

### 2.7. Assessment and correlation analysis of immune infiltrating cell

By utilizing single-sample gene set enrichment analysis (ssGSEA) on 28 immune-related signatures derived from expression profiles, the extent of immune cell infiltration and the characteristics of infiltrating immune cells were investigated. This analysis also uncovered variations in the correlations between the diagnostic biomarker and immune infiltrating cells. Applying the “gsva” package to ssGSEA. Furthermore, the results were visualized utilizing the “ggplot2.”

### 2.8. Cell line and cell culture

Human nucleus pulposus cell (NPC) lines were procured from Shanghai Anwei Biotechnology Co., Ltd. (Shanghai, China). The cells were cultivated in DMEM/F-12 medium (Gibco, United States) supplemented with 10% fetal bovine serum (Gibco, United States) and 1% penicillin/streptomycin (Solarbio, China). The nucleus pulposus cells (NPCs) were maintained at 37 °C in an atmosphere containing 5% CO_2_.

### 2.9. Cell apoptosis model

To establish a NPC apoptosis model, we employed a methodology identical to that of Shen et al (2017).^[32]^ Specifically, cells were initially synchronized by culturing in DMEM/F-12 medium supplemented with 2% fetal bovine serum for 12 hours to mitigate the effects of starvation on autophagy levels. Subsequently, the medium was replaced with either complete medium containing 10% fetal bovine serum or serum-free medium, to which recombinant human interleukin-1β (IL-1β) was added to a final concentration of 10 ng/mL. Cells were then further cultured for 24 hours at 37 °C in a humidified atmosphere of 5% CO_2_ to induce apoptosis.

### 2.10. Western blotting analysis (WB)

Proteins were extracted from the NPCs using RIPA lysis buffer (Beyotime, China). Equal amounts of protein were resolved by 10% SDS-PAGE and subsequently transferred onto a PVDF membrane (BioRad, United States). The membrane was then blocked with 5% nonfat dry milk and incubated with primary antibodies against KCNQ1 (1:1000, Abcam, China) and β-actin (1:2000, Abcam, China) overnight at 4 °C. After washing with TBST, the membrane was incubated with horseradish peroxidase-conjugated secondary antibody (1:2000, Proteintech, China) for 1 hour at room temperature. The immunoblots were visualized using an ECL system (enhanced chemiluminescence) and the band intensities were quantified using ImageJ software.

### 2.11. Ethical review

This study did not require ethical approval as it was conducted using publicly available data and commercially available cell line, without any intervention involving human or animal subjects.

### 2.12. Statistical analysis

R software (version 4.2.3) was used for all data processing and analysis. The Wilcoxon test was used to compare differences between independent and non-normally distributed variables. The coefficients for correlation between distinct genes, as well as the correlation between gene and immune cells, were calculated using Spearman correlation analysis. *P* ≤ .05 was regarded as statistically significant.

## 3. Results

### 3.1. Identification and enrichment analysis of ERS related DEGs in IDD

**Figure 1.**
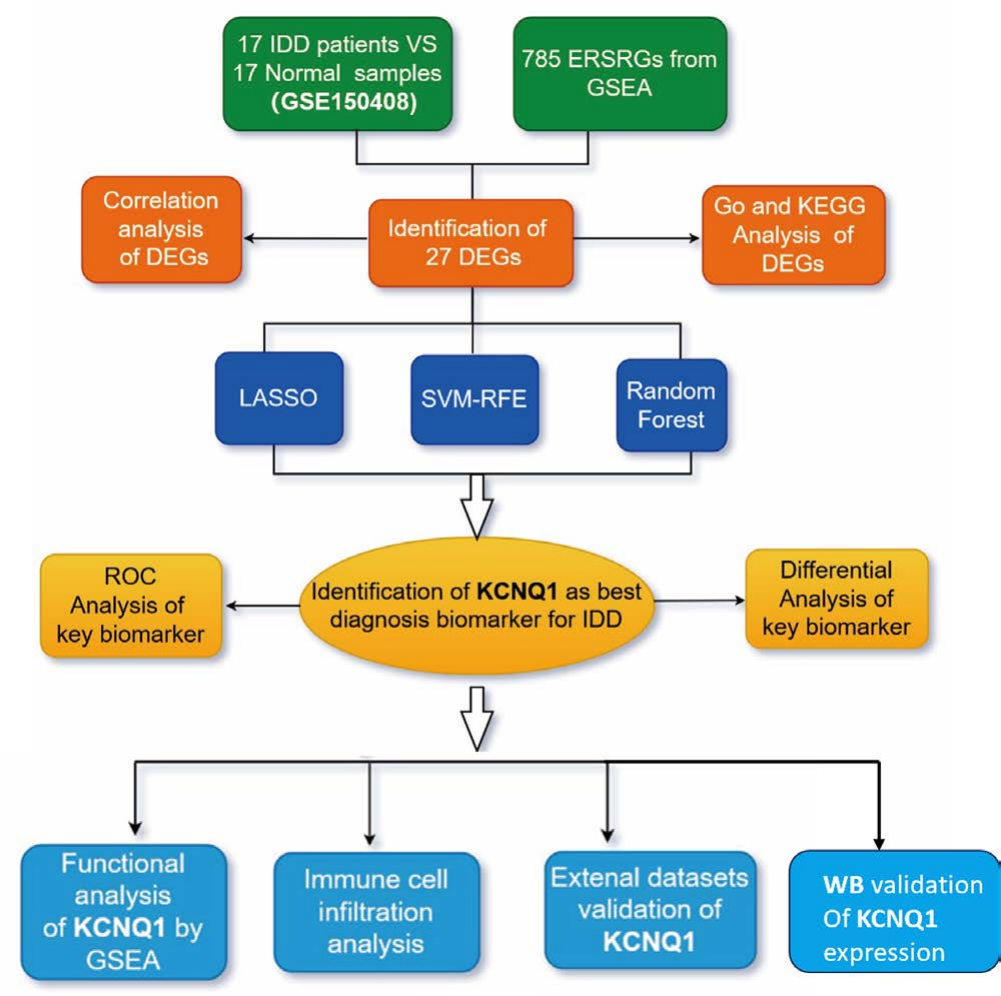
Analysis flow chart.

**Figure 2.**
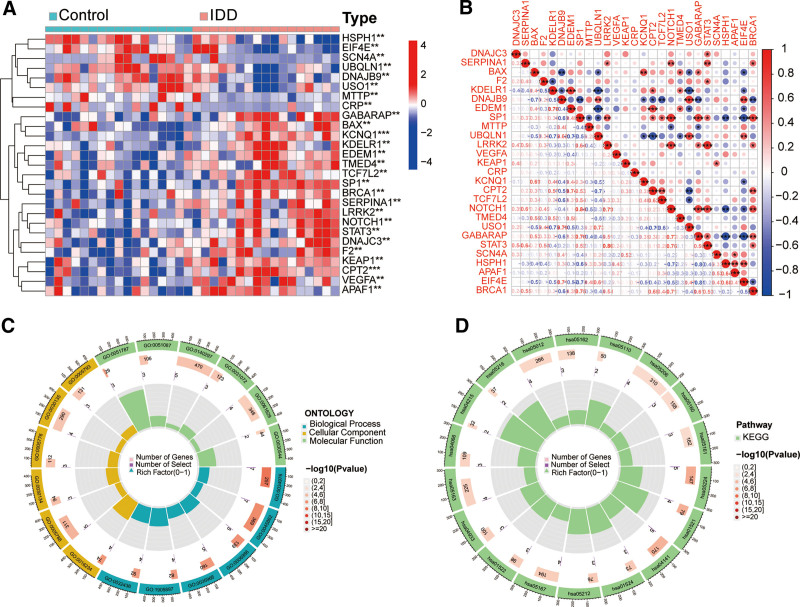
ERS related DEGs and their enrichment analysis. (A) Heatmap plots of ERS related DEGs in IDD. (B) Correlation analysis of ERS related DEGs in IDD. (C) GO functional enrichment analysis of ERS related DEGs. (D) KEGG pathway enrichment analysis of ERS related DEGs. DEGs = differentially expressed genes, ERS = endoplasmic reticulum stress, GO = gene ontology, IDD = intervertebral disc degeneration.

Figure 1 provides a concise overview of the bioinformatics analysis method, illustrating the individual steps involved in this work. In GSE150408, a total of 27 DEGs associated to ERS were identified between patients with IDD and normal controls (Fig. 2A). A robust and noteworthy association was detected among the majority of DEGs in the samples of individuals with IDD (Fig. 2B). The analysis of GO revealed that the DEGs were mostly enriched in processes related to the response to ERS, the endoplasmic reticulum lumen, and DNA-binding transcription factor binding (Fig. 2C). In addition, the KEGG analysis showed a notable increase in DEGs related to protein processing in the endoplasmic reticulum, the HIF-1 signaling pathway, and apoptosis in various species (Fig. 2D).

### 3.2. Identification of potential diagnostic markers via ML

**Figure 3.**
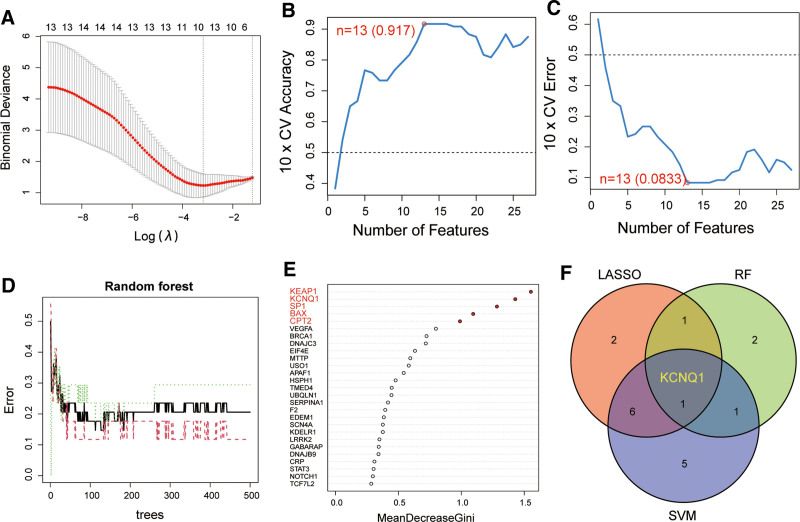
The screening of the diagnostic biomarker. (A) LASSO to screen diagnostic markers for IDD. (B and C) SVM to screen ERS related diagnostic biomarkers for IDD. (D and E) RF to screen ERS related diagnostic biomarkers for IDD. (F) Venn diagram showing KCNQ1 are the best diagnostic gene in IDD. ERS = endoplasmic reticulum stress, IDD = intervertebral disc degeneration, LASSO = least absolute shrinkage and selection operator, SVM = support vector machine.

Using the expression of 27 DEGs associated with ERS, we found 10 potential diagnostic biomarkers for IDD by LASSO (Fig. 3A). Additionally, SVM-RFE analysis revealed 13 potential diagnostic biomarkers for IDD (Fig. 3B and C). Furthermore, the RF algorithm was used to screen the top 5 genes with the highest feature weight (Fig. 3D and E). Through Venn analysis, we concluded that KCNQ1 is the most effective diagnostic biomarker for the development of IDD (Fig. 3F).

### 3.3. Differential expression analysis and ROC curve of KCNQ1 in IDD

**Figure 4.**
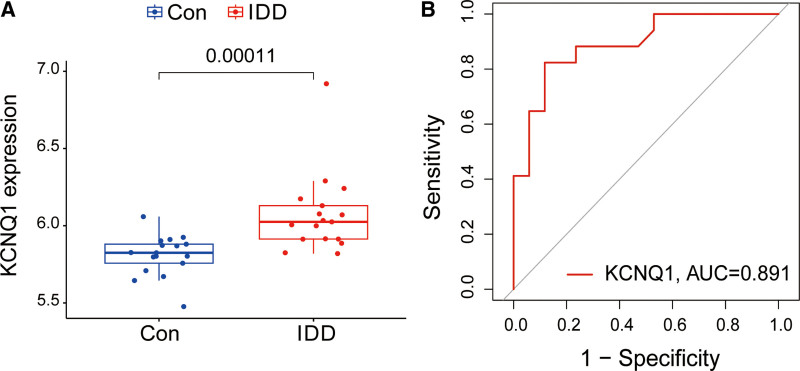
Differential expression analysis and ROC curve of KCNQ1. (A) Differential expression of KCNQ1 in GSE150408. (B) ROC curve evaluating the KCNQ1 diagnostic efficacy in GSE150408. ROC = receiver operating characteristic.

Our analysis revealed a substantial difference in the expression of KCNQ1 between the illness group and the control group. Additionally, based on the ROC curve, KCNQ1 demonstrated promising diagnostic effectiveness. Analysis of differential expression revealed a substantial upregulation of KCNQ1 in IDD (Fig. 4A). The ROC curve analysis showed an AUC of 0.891 for IDD (Fig. 4B).

### 3.4. GSEA of KCNQ1 in IDD

**Figure 5.**
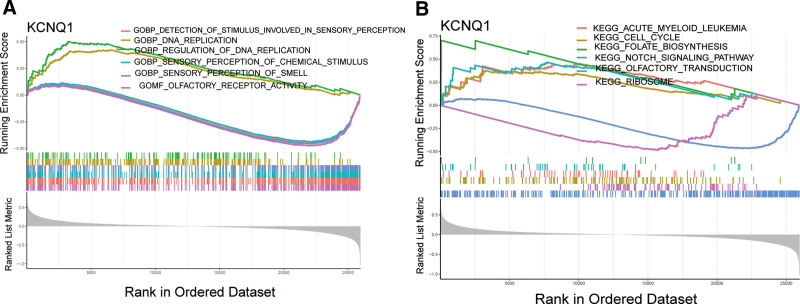
GSEA of KCNQ1 in IDD. (A) GO analysis based on GSEA of KCNQ1 in IDD. (B) KEGG analysis based on GSEA of KCNQ1 in IDD. IDD = intervertebral disc degeneration, GO = gene ontology, GSEA = gene set enrichment analysis.

In the KCNQ1 high-expression group, the GO enrichment analysis based on GSEA of were enriched in DNA replication and regulation of DNA replication (Fig. 5A). The KEGG pathways analysis based on GSEA showed that in the KCNQ1 high-expression group were mainly enriched in cell cycle, folate biosynthesis, and Notch signaling pathway (Fig. 5B).

### 3.5. Immune cell infiltration analysis

**Figure 6.**
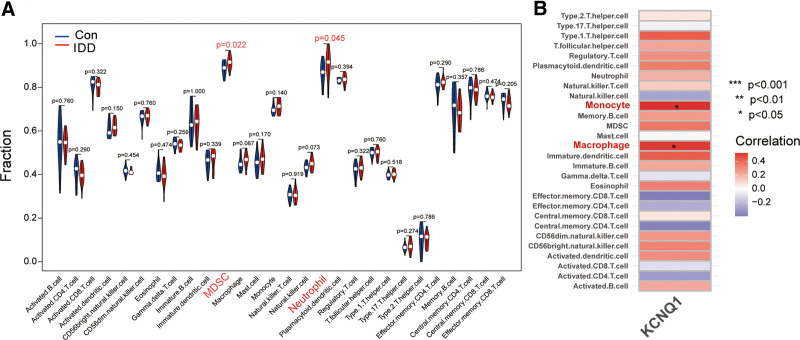
Immune cell infiltration analysis. (A) Immune cell infiltration analysis in GSE150408. (B) Correlation analysis between KCNQ1 and immune cell infiltration in IDD. IDD = intervertebral disc degeneration.

The effect of immune cells on disease was analyzed by comparing differences in immune cell levels between the IDD and control groups in GSE150408 using ssGSEA. In GSE150408, myeloid-derived suppressor cells (MDSC) and neutrophil were higher in IDD samples than in normal control samples (Fig. 6A). In addition, in IDD, KCNQ1 positively regulates the following immune cell infiltrations as shown by correlation analysis between diagnostic biomarker and immune cells: monocyte and macrophage (Fig. 6B).

### 3.6. Verification of KCNQ1 in external datasets

**Figure 7.**
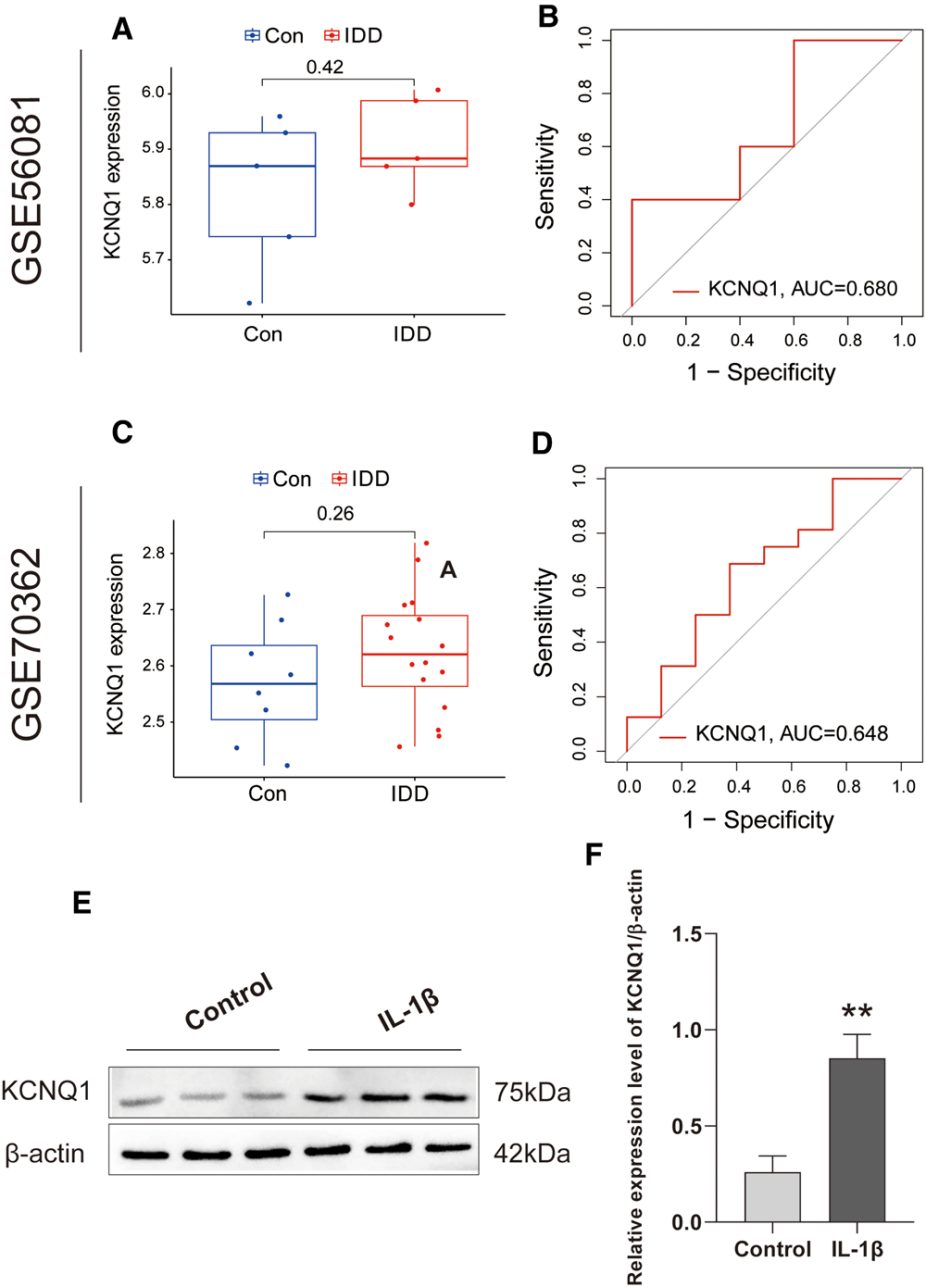
The validation of the KCNQ1. (A) Differential expression of KCNQ1 in GSE56081. (B) ROC curve evaluating the KCNQ1 diagnostic efficacy in GSE56081. (C) Differential expression of KCNQ1 in GSE70362. (D) ROC curve evaluating the KCNQ1 diagnostic efficacy in GSE70362. (E) WB confirmed significantly elevated KCNQ1 protein levels in IL-1β-induced nucleus pulposus cell apoptosis models versus controls. (F) WB quantification showed statistical differences between the 2 groups. ROC = receiver operating characteristic, WB = Western blotting.

In order to provide a more effective demonstration of the diagnostic accuracy of KCNQ1 in IDD, we performed differential expression and ROC curve analysis on KCNQ1 using 2 additional external datasets related to IDD. The analysis of GSE56081 reveals that KCNQ1 has a high level of expression in IDD, which aligns with the expression pattern observed in the test set (Fig. 7A). Furthermore, in GSE56081, the AUC value of KCNQ1 is 0.680, suggesting that this gene exhibits an impressive diagnostic efficacy in IDD (Fig. 7B). It is noteworthy that we also discovered KCNQ1 is significantly expressed in IDD in GSE70362 (Fig. 7C), where its AUC value is 0.684 (Fig. 7D). These results are comparable to those we observed in GSE150408. Figure 7E illustrates a significant increase in KCNQ1 protein expression in the NPC apoptosis model induced by IL-1β as compared to the blank control group. Figure 7F, which is the quantification plot of the WB results, demonstrates a statistically significant difference in KCNQ1 expression between the blank control and IL-1β-treated groups. These integrated outcomes reinforce the diagnostic efficacy of KCNQ1 in IDD and highlight its potential as a biomarker for the condition.

## 4. Discussion

The etiology of IDD is multifaceted and intricate. Maintaining the activities of intervertebral disc and taking part in the synthesis, transport, secretion, and degradation of extracellular matrix-related proteins are crucial roles played by ER.^[33,34]^ However, due to the inadequate understanding of the molecular pathways behind IDD, there are currently no viable treatment options for curing the condition. Since ERS has been shown to be crucial to the onset of IDD, it may be targeted for the therapeutic treatment of the disorder.^[35,36]^ This study employed ML methods that identified the diagnostic biomarker KCNQ1, which is linked to ERS in IDD. Additionally, the study investigated the possible mechanism of KCNQ1 in IDD by GSEA. Furthermore, we conducted an analysis of the regulatory impact of KCNQ1 on the infiltration of immune cells. Our discoveries have the potential to offer valuable implications for individuals with IDD, including the possibility of early detection and monitoring of therapy.

The KCNQ1 gene produces a protein that consists of 6 transmembrane segments. When 4 of these proteins come together, they form a functional hole that selectively allows the passage of potassium ions (K+).^[37]^ Disruption of the human KCNQ1 gene, such as by genetic mutation, is linked to the development of cardiac arrhythmias, specifically atrial fibrillation^[38]^ and long QT syndrome.^[39]^ It is noteworthy that several research have indicated a correlation between KCNQ1 mutation and heightened vulnerability to type II diabetes^[40]^ and gastric cancer.^[41]^ Furthermore, the elimination of the Kcnq1 gene in mice results in hearing loss, alterations in insulin responsiveness, and the absence of stomach acid production.^[42,43]^ Currently, there is a lack of research on the involvement of KCNQ1 in IDD. However, research has proposed that KCNQ1OT1, a LncRNA associated with KCNQ1, plays a crucial role in IDD.^[44]^ Our findings revealed that KCNQ1 potentially contributes to the development of IDD via the Notch signaling pathway, as indicated by GSEA. Previous studies has indicated that the Notch signaling pathway is crucial in preserving the integrity of intervertebral discs. Long et al discovered that injecting recombinant JAG2 can lessen the degenerative changes in nucleus pulposus by increasing the levels of ECM components and decreasing the levels of catabolic factors. On the other hand, injecting lentivirus sh Notch2 can make the degenerative changes in NP worse.^[45]^ Furthermore, it has been demonstrated that Notch signaling reduces NF-κB-induced inflammatory responses in the intervertebral disc.^[46]^ In addition, the hypoxic condition in IDD can maintain the dynamic stability of the cell population by controlling the activation of the Notch signaling pathway.^[47]^ In summary, Notch signaling transduction plays an important role in regulating the proliferation and apoptosis of NPCs and annulus fibrosus cells in intervertebral disc, thereby indirectly regulating the progression of IDD.^[47]^ As a result, we believe that KCNQ1 may play a significant part in the development of IDD by way of the Notch signaling pathway, and that KCNQ1 may be a potential crucial target for the prevention and treatment of IDD.

As the biggest avascular organ in the body, intervertebral discs are immune privileged organs that are protected from immunological and inflammatory factor infiltration.^[48,49]^ Our research has found that KCNQ1 positively controls monocyte and macrophage infiltration according to the correlation study of KCNQ1 and immune cell infiltration. This is in line with other research findings, which show that immune cells, particularly monocytes and macrophages, produce reactive oxygen species that can precipitate the onset of oxidative stress, leading to severe metabolic disorders and even cell death. Reactive oxygen species can also trigger pro-inflammatory factors and transcription factors, which can speed up the inflammatory response.^[50]^ As a result, KCNQ1 is crucial in encouraging the infiltration of monocytes and macrophages into intervertebral discs, which advances IDD.

While ML algorithms have found ERS related diagnostic biomarker of IDD, external datasets have proven the diagnostic efficacy. Nevertheless, the Gene Expression Omnibus database’s expression data and fundamental sample characteristics were the only things examined in this study. Age, the Pfirrmann grade of the IDD, the disc level involved, and the type of IDD (disc herniation, degenerative spondylolisthesis, for example) were among the more specific clinical data that we were unable to obtain. As a result, we were unable to rule out additional confounding factors that might have an impact on KCNQ1 expression. We plan to validate our findings in the future by creating prospective cohort studies that gather more detailed and multifaceted data. Furthermore, more experimental validation is needed to fully comprehend KCNQ1’s function and any potential regulatory mechanisms in IDD using methods like immunohistochemistry and RT-qPCR.

## 5. Conclusions

In this work, we utilize ML approaches to discover KCNQ1 as a diagnostic biomarker for IDD that are connected to ERS. Through GSEA research, it was discovered that KCNQ1 primarily contributes to IDD by influencing the Notch signaling pathway. Additionally, study of the correlation between KCNQ1 and immune cells revealed that KCNQ1 regulates the invasion of monocytes and macrophages to promote IDD. Finally, by using IL-1β to construct an NPC apoptosis model, WB showed that KCNQ1 was significantly upregulated in the apoptotic group. We will perform more in vitro and in vivo tests to better explore the underlying causes of IDD, building upon the insights provided by our study.

## Acknowledgments

The data provided by participants in GEO, including patients and researchers, is sincerely appreciated by the authors.

## Author contributions

**Conceptualization:** Yongquan Huang, Feng Wu.

**Data curation:** Feng Wu.

**Formal analysis:** Feng Wu.

**Investigation:** Xin Hu, Feng Wu.

**Methodology:** Xin Hu.

**Project administration:** Yongquan Huang, Xin Hu.

**Resources:** Yongquan Huang.

**Supervision:** Yongquan Huang.

**Software:** Xing Li.

**Writing – original draft:** Xing Li, Xin Hu, Feng Wu.

**Writing – review & editing:** Xing Li, Feng Wu.
